# Analysis of facial expressions in response to basic taste stimuli using artificial intelligence to predict perceived hedonic ratings

**DOI:** 10.1371/journal.pone.0250928

**Published:** 2021-05-04

**Authors:** Takashi Yamamoto, Haruno Mizuta, Kayoko Ueji

**Affiliations:** 1 Health Science Research Center, Kio University, Nara, Japan; 2 Department of Nutrition, Faculty of Health Sciences, Kio University, Nara, Japan; National Institutes of Health, UNITED STATES

## Abstract

Taste stimuli can induce a variety of physiological reactions depending on the quality and/or hedonics (overall pleasure) of tastants, for which objective methods have long been desired. In this study, we used artificial intelligence (AI) technology to analyze facial expressions with the aim of assessing its utility as an objective method for the evaluation of food and beverage hedonics compared with conventional subjective (perceived) evaluation methods. The face of each participant (10 females; age range, 21–22 years) was photographed using a smartphone camera a few seconds after drinking 10 different solutions containing five basic tastes with different hedonic tones. Each image was then uploaded to an AI application to achieve outcomes for eight emotions (surprise, happiness, fear, neutral, disgust, sadness, anger, and embarrassment), with scores ranging from 0 to 100. For perceived evaluations, each participant also rated the hedonics of each solution from –10 (extremely unpleasant) to +10 (extremely pleasant). Based on these, we then conducted a multiple linear regression analysis to obtain a formula to predict perceived hedonic ratings. The applicability of the formula was examined by combining the emotion scores with another 11 taste solutions obtained from another 12 participants of both genders (age range, 22–59 years). The predicted hedonic ratings showed good correlation and concordance with the perceived ratings. To our knowledge, this is the first study to demonstrate a model that enables the prediction of hedonic ratings based on emotional facial expressions to food and beverage stimuli.

## Introduction

Taste is one of the most important sensations for determining the pleasantness and acceptability of food and beverages. The taste components of food and beverages generally consist of different combinations of six basic tastes with distinct qualities: sweet, salty, sour, bitter, umami, and fatty [1, 2]. These components also elicit different hedonics, ranging from extremely pleasant (by sweet stimuli) to extremely unpleasant (by bitter stimuli). Therefore, the pleasantness and acceptability of food and beverages may be closely correlated with taste, in addition to the importance of odor and/or texture. Conventional self-report questionnaires and sensory tests using a visual analog scale are common and useful for evaluating the hedonics of food, beverages, and taste. However, scores tend to vary depending on the personal opinions of judges, previous experiences, and factors such as internal and external conditions [3, 4]. Therefore, efficient objective methods have long been desired for the hedonic evaluation of food and beverages.

For this purpose, various physiological reactions evoked by taste stimuli depending on the quality and/or hedonic tone have been more or less used for the objective evaluation of food and beverage hedonics (e.g., positive vs. negative; palatable vs. aversive; pleasant vs. unpleasant; acceptable vs. rejective). For example, taste effects can be observed in autonomic nerve activity such as salivary secretion [5, 6], heart rate [7, 8], and facial blood flow [9, 10], hormonal changes such as insulin [11] and cortisol secretion [12], motor reactions such as facial expressions [13–17] and bodily reactivity [17], and brain activity [18–21]. A recent study from our lab revealed that pleasant and unpleasant edibles tended to elicit decreased and increased oxyhemoglobin (oxyHb) levels, respectively, within the ventral part of the anterior prefrontal cortex, suggesting that monitoring of oxyHb in this region may prove useful for the objective evaluation of food and beverage hedonics [21]. Although this technique could be efficiently utilized, it is available only for those who can use such sophisticated equipment to measure oxyHb in the cerebral blood vessels. The purpose of this study was to develop a more convenient and accurate objective method to evaluate the hedonics of food and beverages in practical situations.

Also in this study, from among the possible approaches for the objective evaluation of the hedonics of taste, we targeted facial expressions because these can be easily and practically monitored without the need to use any particular equipment or devices for bioelectric measurements or humoral analyses. Moreover, previous studies have suggested that facial expressions reflect the hedonic aspects of taste. For example, in his pioneering studies [13–16], Steiner revealed that facial expressions to taste stimuli were expressed dominantly by hedonic tone as opposed to the qualitative aspects of taste. Since such facial expression patterns could be observed in not only normal subjects, including neonates, but also in abnormal subjects, including anencephalic and hydroanencephalic neonates, he termed taste-induced facial expressions a “gustofacial reflex” not requiring previous experience. In both humans and animals, orofacial and bodily responses are induced by taste stimuli, i.e., taste-induced motor reactions have been used as a taste reactivity test to monitor the hedonics of taste stimuli in animals [16, 17]. Although such taste-elicited motor reactions are useful, video analyses of movements are very time-consuming because to verify and count the frequency of occurrence of characteristic motion features belonging to positive or negative hedonics, trained researchers have to carry out frame-by-frame observation [15, 17, 22].

Therefore, in this study, we aimed to analyze facial expressions with the aid of artificial intelligence (AI) technology to examine whether facial expressions can be used as a method for rating food and beverage hedonics. Essentially, the same approach has recently been assessed by several researchers in different fields of food research [3, 4, 22–28]. Most researchers have used an automated facial expression recognition system (computer software with built-in information about changes in human facial expressions to different emotions) such as FaceReader (Noldus Information Technology, Wageningen, The Netherlands) to analyze facial expressions in response to various food and beverage stimuli under different experimental conditions using basic expressions of human emotions, such as happiness, anger, sadness, and disgust.

However, the utilization of facial emotions is very limited. Previous studies [3, 4, 7, 22, 24, 27] have simply compared the score of each emotional component individually with explicit self-reported hedonic ratings for food and beverages. Facial expressions are induced in a spontaneous manner by unconscious mechanisms, whereas explicit self-reported hedonic ratings are subjective and easily biased by many influencing factors; therefore, we would like to expand the role of facial expressions from just one of the objective indicators of the hedonics of food and beverages to a method that can enable accurate ratings between extremely unpleasant and extremely pleasant (e.g., –10 vs. +10, respectively), comparable to the subjective self-reported hedonic ratings. For this purpose, taking advantage of an AI tool that analyzes and classifies facial expressions into several basic emotions, we devised a regression equation by combining the score of each emotion evoked by five basic taste stimuli with wide hedonic valence ranging from extremely unpleasant to extremely pleasant. Using this equation, we can predict hedonic ratings (between –10 and +10) for new taste substances. Our hypothesis is that the obtained formula can be a standard tool that enables the accurate prediction of hedonic ratings for any food or beverage in every person. We examine the applicability of the formula using the emotion scores to another taste solutions obtained from other participants of both genders in different age groups. If such a tool could be developed, it might be expected to be utilized, for example, as a convenient sensory test to compare taste hedonics among various products, as a consumer affective test for new food products before their introduction, and as a tool for those who have difficulty expressing subjective ratings.

Since this study is a new and first step in predicting hedonic ratings from facial expressions, the hypothesis is only tested for several commercially available beverages and five basic taste solutions in a limited number of persons of both genders and in different age groups. Food was not used in this study as a test sample to avoid disturbing facial expressions, which may generally occur during the chewing and eating of solid food.

## Materials and methods

### Participants

A total of 22 nonsmoking participants were recruited from among the students and staffs of Kio University in Nara, Japan. Based on the responses to a questionnaire conducted before the experiment, we judged that all participants were free of sensory, eating, neurological, and psychiatric disorders, and none were using any medications that would interfere with taste. All participants were instructed to refrain from eating or drinking from 1 hour before the start of the experiment. After providing an explanation of the purpose and safety of the experimental protocol, written, informed consent was obtained from all participants. This study was approved by the Kio University ethics committee (No. H27-15), and all experiments were conducted in accordance with the principles set forth in the Declaration of Helsinki.

### Experiment 1

A cohort of 10 healthy female volunteers (age range, 21–22 years) were recruited from among students at Kio University to participant in an experiment to examine the utility of AI for the analysis of facial expressions and establish a formula to predict hedonic ratings. One session of the experiment was operated by three persons: two researchers in our lab as experimenters and one participant. The experimenters prepared taste solutions with water (distilled water, DW) that consisted of nine kinds of five conventional basic tastes with different concentrations: 2.5%, 5%, 10% and 20% sucrose; 0.5% and 2% monosodium glutamate [MSG]; 1% citric acid; 5% sodium chloride [NaCl]; and 0.02% quinine hydrochloride [QHCl]. One experimenter (the “deliverer”) put 10 mL of taste solution or DW in a small paper cup and placed it on a table just in front of the participant, who was sitting in a quiet room. The deliverer asked the participant show facial expressions freely but not intentionally, and to make a brief comment about the quality and/or palatability of the stimulus soon after recognition (e.g., “this taste is sweet and very good”). The participant poured the 10 mL of liquid into her mouth, held it for about 1 second, and then swallowed. After the participant drank the liquid and rinsed her mouth well with DW, the deliverer placed another cup on the table, and the task was repeated. The inter-stimulus interval was at least 2 minutes. Each of the 10 stimuli (one DW and nine taste solutions) was delivered randomly. The participant was also asked to evaluate the overall hedonic rating of the stimulus on a scale from ‒10 (extremely unpleasant) to +10 (extremely pleasant), with 0 being neutral, before the start of the next tasting.

Another experimenter (the “recorder”) sat near the participant, gave the signal to start drinking, and recorded a video focusing on the face of the participant from the start of drinking to about 10 seconds after swallowing using a smartphone camera (iPhone; Apple Inc. Cupertino, CA). The camera was set 2 m in front of the participant, who was asked to look directly at the camera and remove the cup immediately after swallowing to avoid covering her mouth. This experimenter then saved the participant’s comments and self-reported hedonic rating for the stimulus in a personal computer. After the task, the deliverer informed the recorder about the order and names of the delivered stimuli. This task was then repeated for the remaining nine participants.

After the experiment, a frame (or a single image) from within a few seconds before or after the participant’s comment in the video replay was selected by the two experimenters. This image was then loaded into a freely available AI application (*Kao-shindan*, Japanese for “face diagnosis”; API version 1.7, developed by MONOPOLEAPPS K.K. in 2018; https://apps.apple.com/jp/app/id1267719377) installed on the smartphone for the analysis of facial expressions. After uploading the image, the AI application classified the facial expression for eight different emotions (surprise, happiness, fear, neutral, disgust, sadness, anger, and embarrassment), with scores ranging from 0 (no visible emotion) to 100 (emotion is fully present) for each. This procedure is similar to that used in Microsoft Face API (https://azure.microsoft.com/en-us/services/cognitive-services/face/), which analyzes emotions in images. The recorder then saved the scores for each of the eight different emotions classified by the AI for each stimulus in the computer.

In the next step, a multiple linear regression analysis was performed to predict hedonic ratings based on the eight emotions (predictors). The calculation was based on the scores of the eight emotions obtained to 10 stimuli in 10 persons and the participants’ perceived (self-reported) hedonic ratings for each stimulus.

### Experiment 2

Another cohort of 12 participants (6 males, 6 females; age range, 22–59 years) from among Kio University students and staff was recruited for a second experiment, the purpose of which was to examine and confirm the applicability of the formula obtained in Experiment 1 for predicting hedonic ratings. None of the participants in Experiment 2 had participated in Experiment 1. Except for the DW, 10 stimuli used in Experiment 2—i.e., 15% glucose, 1% NaCl, 50% lemon juice (Pokka Lemon 100; Pokka Sapporo Food & Beverage, Ltd., Tokyo, Japan), 0.003% sucrose octaacetate as bitter taste, 0.5% inosine monophosphate as umami taste, 1.5% instant coffee (Nescafe Gold Blend; Nestle Japan S.A., Kobe, Japan), miso soup (Tokujyo; Takeya-Miso Co., Ltd., Suwa, Japan), 1% bonito soup stock (Hon-dashi; Ajinomoto Co., Inc., Tokyo, Japan), catechin jasmine tea (Ito-En, Ltd., Tokyo, Japan), and apple juice (Dole Japan, Inc., Tokyo, Japan)—were different from those used in Experiment 1. NaCl was also used, but the concentration was different from that used in Experiment 1.

Liquid intake, picture taking, AI face analysis, and the rating of perceived hedonics were the same as those used in Experiment 1. AI outputs for the emotions of facial expressions in response to these stimuli were put in the corresponding emotions in the formula used in Experiment 1 to obtain predicted (or calculated) hedonic ratings. The relationships between the predicted and perceived hedonic ratings were then examined and compared.

### Data analysis

In Experiment 1, the boxplot analysis of the scores for the eight emotions associated with each of the 10 stimuli in 10 participants was conducted, and the median and interquartile range with minimum and maximum scores were obtained. To examine the similarity of hedonics among taste stimuli, Spearman’s correlation coefficients were calculated between pairs of stimuli based on the scores of eight emotions in 10 participants. A multiple linear regression analysis was then conducted based on the scores for the eight emotions (predictors) obtained to 10 stimuli in 10 participants and the participants’ perceived hedonic ratings for each stimulus. The possible existence of multicollinearity, which occurs when predictors provide redundant information because of a high correlation with each other, was examined by calculating the correlation coefficients among pairs of eight emotions. When a statistically significant positive correlation was detected between any pair of emotions, that pair was combined, and the mean score was used. In Experiment 2, relationships between the predicted and perceived hedonic ratings were examined and compared using Pearson’s and Spearman’s correlation coefficients, intraclass correlation coefficients, Friedman test, and Wilcoxon signed-rank test. Before correlation analyses, data were checked if they showed normal distribution or not by Shapiro-Wilk’s test. All statistical analyses were performed using IBM SPSS Statistics (ver. 25). *P* values < 0.05 were considered statistically significant.

## Results

**Fig 1:**
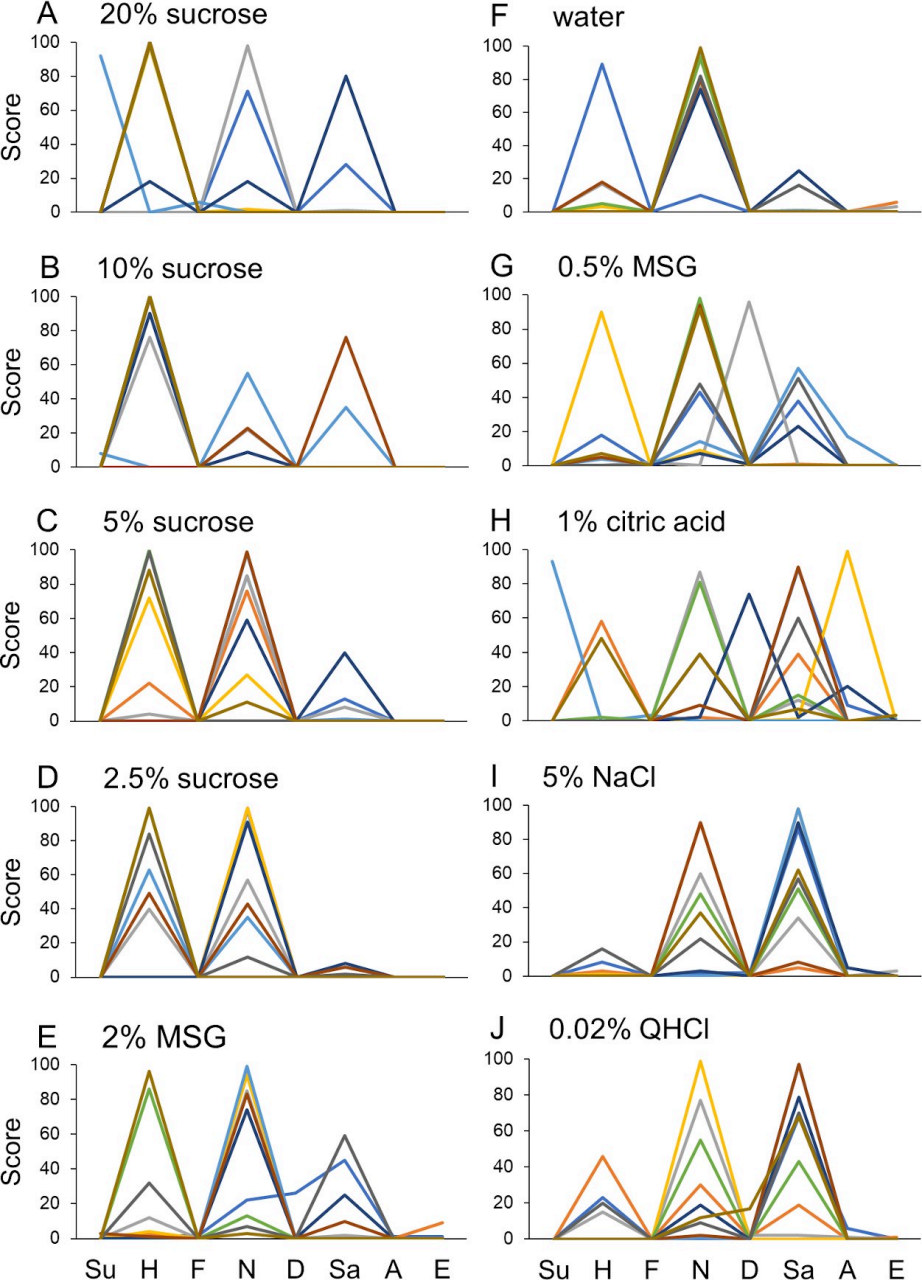
Profiles of hedonic patterns across eight different emotions based on Artificial Intelligence (AI) analysis of facial expressions in response to 10 stimuli in 10 participants. Profiles are depicted in different colors for the 10 participants. The emotions are arbitrarily arranged from left to right in the order of surprise (Su), happiness (H), fear (F), neutral (N), disgust (D), sadness (Sa), anger (A), and embarrassment (E).

**Fig 2:**
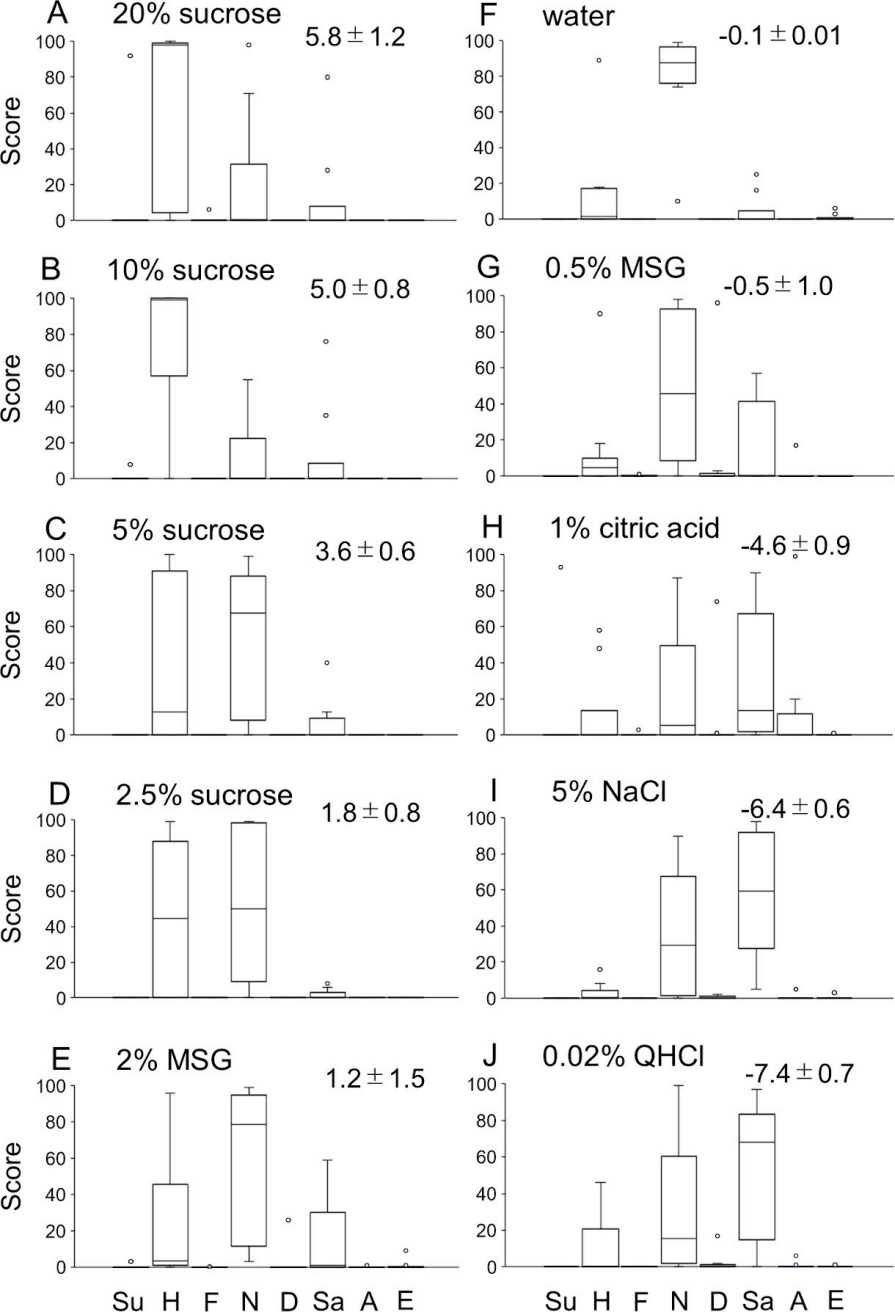
Box and whisker plot analysis of scores for eight different emotions evoked by 10 stimuli. The median and interquartile range with minimum and maximum scores are based on the emotional scores of the 10 participants shown in Fig 1. Small circles in each panel indicate outliers. Graphs are arranged from the most pleasant to the most unpleasant stimulus from A to J, respectively, as shown by the mean ± standard error perceived hedonic rating in each graph. Other descriptions are the same as those in Fig 1.

The scores for eight emotions from the AI outputs associated with facial expressions induced after the presentation of taste stimuli varied among the participants. Fig 1 shows the hedonic pattern profiles across eight different emotions in response to 10 taste stimuli in 10 participants. Fig 2 shows the boxplot analysis of the scores for the eight emotions shown in Fig 1. The median and interquartile range with minimum and maximum scores are illustrated for the emotions evoked by the 10 stimuli. The mean perceived hedonic rating ± standard error for each stimulus in 10 participants is also indicated in each graph. The profiles of scores for the eight emotions suggested that higher concentrations (20% and 10%) of sucrose showed a strong happiness component, whereas the sadness component was the largest for 1% citric acid, 5% NaCl, and 0.02% QHCl; the neutral component was the largest for the remaining stimuli.

**Fig 3:**
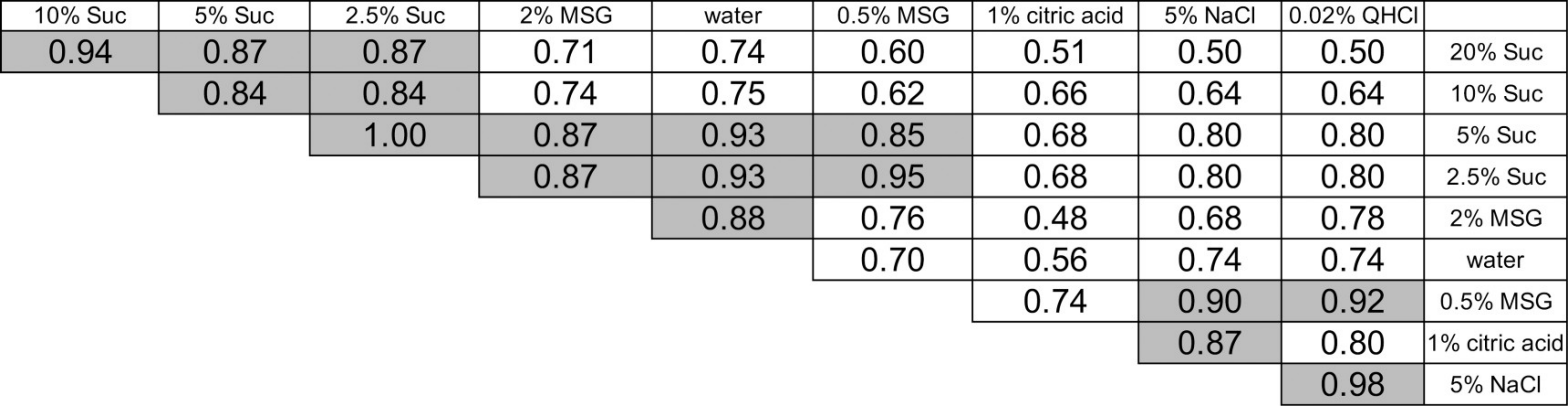
Spearman’s correlation coefficient matrix of the hedonic profiles for 10 stimuli. Correlations were calculated between pairs of stimuli based on the mean scores of eight emotions shown in Fig 1. The taste stimuli were arranged in the order of the most positive to most negative perceived hedonic ratings from left to right and from top to bottom. Coefficients shaded are statistically highly significant (*P* < 0.01).

Fig 3 shows the calculated correlation coefficients among profiles of emotional scores for 10 stimuli. We used Spearman’s analysis because scores did not show normal distribution in some of the stimuli. Statistically significant correlations were detected in three groups. The first group consisted of 20%, 10% and 5% sucrose (Fig 2A to 2C, respectively) with highly positive perceived hedonic ratings. The second group consisted of 2.5% sucrose, 2% MSG and water (Fig 2D to 2F, respectively) with slightly positive or neutral ratings. The third group consisted of 0.5% MSG, 1% citric acid, 5% NaCl, and 0.02% QHCl (Fig 2G to 2J, respectively) with negative hedonic ratings.

A multiple linear regression analysis was performed to predict hedonic ratings based on the scores for the eight emotions obtained in Experiment 1. Before the analysis, multicollinearity was ensured by examining the correlation coefficients among the eight emotions. The only statistically significant (*P* < 0.01) correlation was for the pair of “surprise” and “fear”, so we combined the two emotions into the predictor of “surprise/fear”. As a result, we obtained the following regression formula [F(7, 92) = 12.641, *P* < 0.000 with an adjusted R^2^ of 0.451]:

Hedonic rating = 0.024 × surprise/fear + 0.138 × happiness + 0.097 × neutral + 0.055 × disgust + 0.021 × sadness + 0.031 × anger– 0.584 × embarrassment– 8.943

where “surprise/fear” denotes the mean score of surprise and fear. We found that “happiness” was a significant (*P* = 0.015) predictor of hedonic ratings, while “surprise/fear” (*P* = 0.057), “neutral” (*P* = 0.087), and “embarrassment” (*P* = 0.086) were marginally significant predictors.

**Fig 4:**
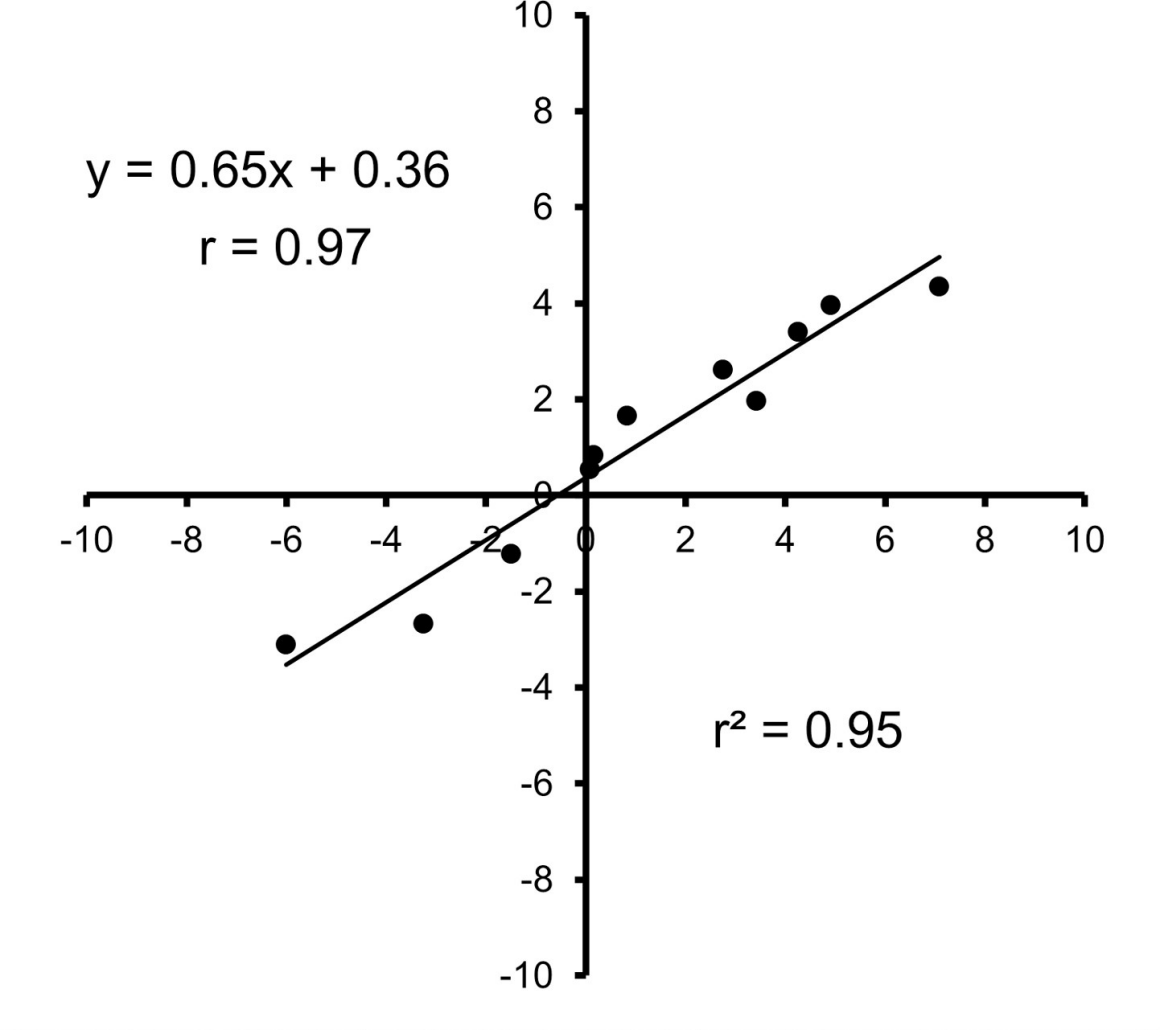
Scatterplots between mean predicted (objective) and perceived (subjective) hedonic ratings in response to 11 stimuli in 12 participants (group analysis). The mean calculated hedonic ratings are shown in the ordinates and the perceived hedonic ratings in the abscissas. The Pearson’s correlation coefficient between the two ratings was very highly significant (*P* < 0.001).

The validity of this formula was examined by applying the obtained emotion scores to another set of taste stimuli in another group of participants who had not been associated with the formula in Experiment 1. First, we applied a group analysis, i.e., the correlation analysis was performed between the mean predicted and perceived ratings to taste stimuli in 12 participants. We used Pearson’s correlation analysis since we found that the data for the calculated and perceived data showed a normal distribution (Kolmogorov-Smirnov’s test). The correlation coefficient was 0.974 (*P* < 0.001) as shown in Fig 4. Since Pearson’s correlation analysis does not directly show the degree of coincidence between predicted and perceived ratings, we used an analysis of the intraclass correlation coefficient (ICC) to assess the consistency, or conformity, of the two ratings. We found that the ICC(2,1) was 0.907 (*P* < 0.001), indicating that both ratings were in good concordance.

**Fig 5:**
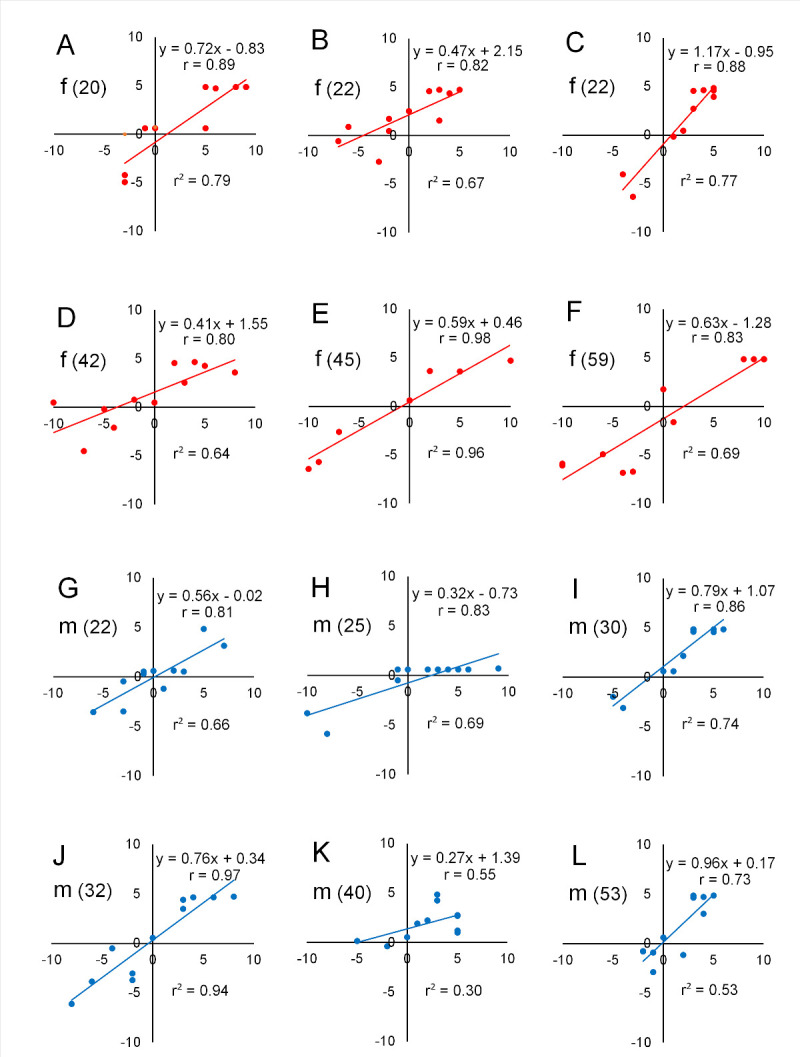
Scatterplots between predicted and perceived hedonic ratings in response to 11 stimuli in 12 participants (single case analysis). Six females (f), depicted in red, are shown in A to F, and six males (m), depicted in blue, are shown in G to L, from younger to older, with ages shown in parentheses. The calculated hedonic ratings are shown in the ordinates and the perceived hedonic ratings in the abscissas. The regression formula, Spearman’s correlation coefficient (r), and coefficient of determination (r^2^) are shown in each graph. *P* < 0.001 for A, C, E, I and J; *P* < 0.01 for B, D, F, G, H and L; and *P* > 0.05 for K.

How the estimated ratings are correlated with the perceived ratings in each person is very important to examine for practical use of the present method in the field of the sensory evaluation of food and beverages. We calculated Spearman correlation coefficients for each participant since the data did not show a normal distribution in some participants. The predicted (or calculated) hedonic ratings were statistically significantly correlated with perceived hedonic ratings in 11 of the 12 females and males with varying ages (Fig 5). One participant, a 40-year old male, did not show significant correlation as shown in Fig 5K (correlation coefficient = 0.545, *P* = 0.076).

**Fig 6:**
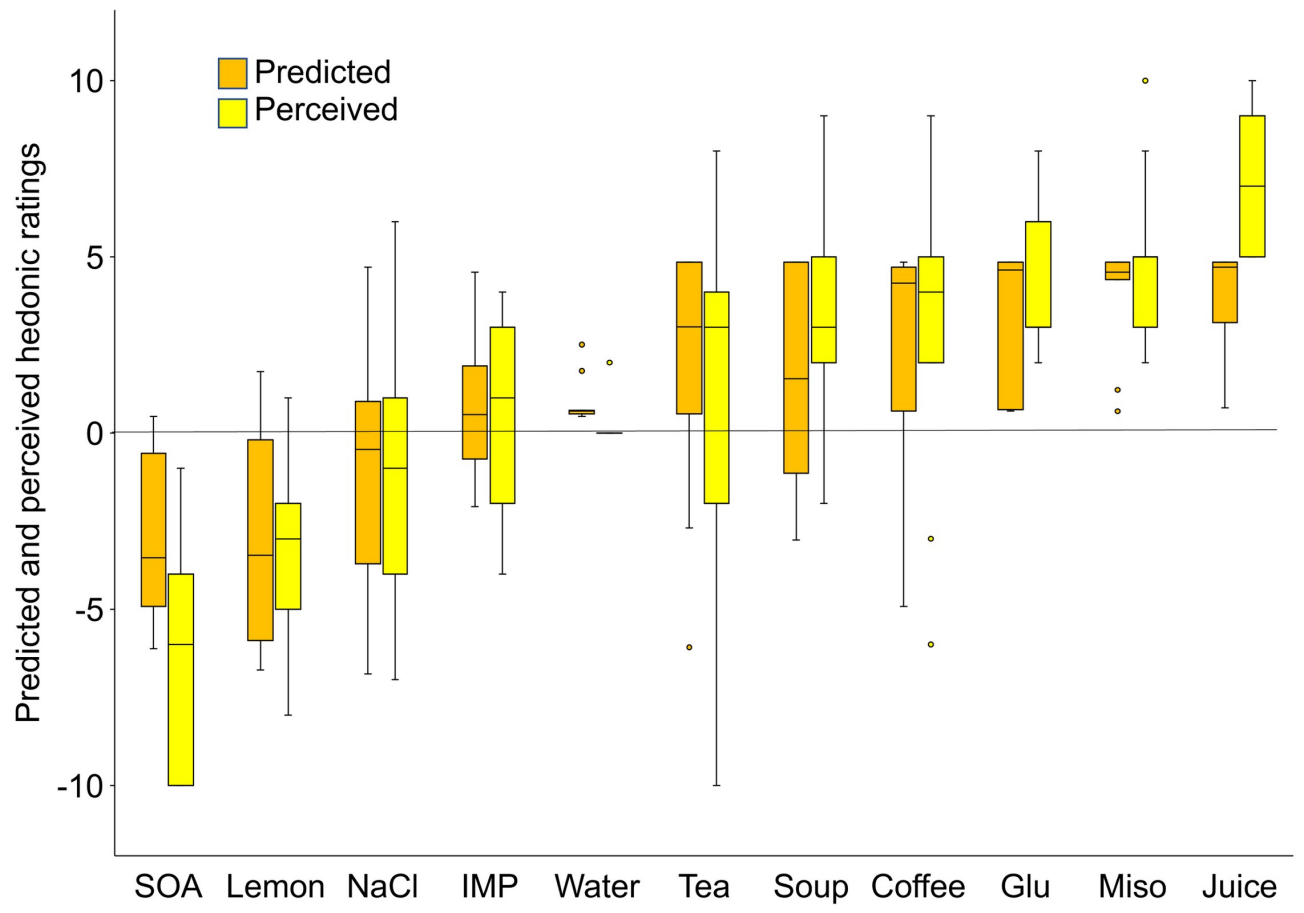
Box and whisker plot analysis of predicted and perceived ratings for 11 stimuli. The median and interquartile range with minimum and maximum scores are based on the hedonic scores of the 12 participants. Small circles in each panel indicate outliers. Graphs are arbitrarily arranged from the most unpleasant to the most pleasant stimulus from left to right. Juice, apple juice; Miso, miso soup; Glu, 15% glucose; Coffee1.5% instant coffee; Soup, 1% bonito soup stock; Tea, catechin jasmine tea; Water, distilled water; IMP, 0.5% inosine monophosphate; NaCl, 1% sodium chloride; Lemon, 50% lemon juice; SOA, 0.003% sucrose octaacetate.

In addition to these correlation analyses, we examined the concordance of the predicted and perceived ratings for each taste stimulus in a group of 12 participants. We show box and whisker plots in Fig 6 for the two ratings in an arbitrary order from the most unpleasant to the most pleasant stimuli. The difference of medians between the estimated and calculated ratings for each taste stimulus could be an indicator as to whether both ratings agree well. When calculated, the mean difference of medians for all 11 stimuli was 0.966, which corresponds to 4.83% of the total length of the sensory scale (20: from –10 to +10), suggesting good agreement between the two ratings. Statistical analysis for the groups of predicted and perceived ratings showed a significant inter-stimulus difference (Friedman test, *P* < 0.001), but no difference between predicted and perceived ratings (Friedman test, *P* = 0.763). However, a significant difference was detected between the two ratings for very palatable apple juice and very aversive sucrose octaacetate (SOA) (Wilcoxon signed-rank test, *P* < 0.01). Such a difference between the two ratings for SOA and apple juice may have been due to the limitation of the predicted ratings on reaching the maximum hedonic ratings, such as –10 or +10.

## Discussion

The major findings of the present study can be summarized as follows. First, we were able to classify five basic tastes into the three categories of hedonically positive, neutral, or negative based on AI analysis of facial expressions in single images. Second, we established a formula that could predict hedonic ratings using multiple linear regression analysis based on emotional facial expressions in response to basic taste stimuli in female students. Third, when we input scores for emotional facial expressions in response to different tastants in different participants of both genders with different ages in this formula, we found a good correlation and concordance between predicted (or calculated) and perceived (or subjective) hedonic ratings. To our knowledge, this is the first study to demonstrate that a single image of a person’s face can easily predict on a quantitative scale the degree to which that person is enjoying food and beverages.

Our finding that facial expressions are related to hedonic aspects of taste is essentially the same as that of the human “gustofacial reflex” reported by Steiner [13–16] and “taste reactivity” in rats by Grill and Norgren [17]. According to those studies, although some characteristic motion features of the face (or motion components of facial reactions) occur depending on the quality of the taste, more dominant facial expressions represent a hedonic distinction between whether stimuli are acceptable or aversive. Acceptable stimuli are pleasant, palatable and nutritive, whereas aversive stimuli convey warning messages in the form of discomfort, harm, and urgency, which are the fundamental bases of food selection and taste function among consumers. To reach a scientific conclusion, Grill and Norgren [17] had to exert time-consuming efforts to observe the frequency of facial motion features using slow-motion or frame-by-frame video playback.

Recently, however, researchers [3, 4, 7, 22–28] have used much more convenient and accurate automated facial expression recognition systems, including sophisticated artificial network systems such as FaceReader or iMotions software (iMotions, Inc., Copenhagen, Denmark), which can classify facial expressions into the following basic universal human emotions suggested by Ekman and Friesen [29], with intensity ranging from 0 to 1: happy, sad, angry, surprised, scared, disgusted, and neutral. Analyses of these emotions are utilized effectively in various experimental situations in food research, e.g., a time course of changes in each emotion after tasting breakfast drinks [7], a comparison between implicit (spontaneous) and explicit (intentional) facial expressions after tasting different juices [22], a comparison between before and after sensory-specific satiety [24], a correlation between hedonic liking and facial expressions [27], a comparison of facial expressions in response to different tastes between persons with and without depressive disorders [4], and differences in responses to various food stimuli between Asians and Western populations [3, 26]. Most of these previous studies indicate the score of each emotional component or the valence that indicates whether the person’s emotional status is positive or negative by calculating the score of happiness minus that of negative emotions such as sadness, anger, fear, and disgust [4].

In the present study, we used a freely available AI application for reading facial expressions (*Kao-shindan* API, version 1.7). This application can classify facial expressions into eight different emotions with intensity scores ranging from 0 to100 for each based on a single image within 10 seconds. This software is similar to Microsoft Face API, which also analyzes emotions in a single image. Even with the use of such a simple method, we could obtain the characteristic profile of emotional facial expressions to each of the basic taste stimuli with different concentrations for sucrose and MSG (see Figs 1 and 2).

Concerning the use of such facial emotions, most previous studies have correlated the score of each emotional component separately with the hedonic valence of food and beverages, as listed above. However, since we were interested in the use of facial expressions as a practical tool for the objective evaluation of the overall hedonics of food and beverages, we hypothesized that we could predict hedonic ratings through a comprehensive treatment of all the emotions together. For this purpose, we developed a formula capable of predicting hedonic ratings. Specifically, multiple linear regression analysis was conducted based on perceived hedonic ratings and eight emotional facial expressions (predictors) in response to 10 stimuli, including five basic tastes, and water in 10 female students aged 21–22 years. The validity of the formula was ascertained by applying the emotion scores obtained to another set of stimuli, including basic taste solutions, and commercially available beverages in another 12 participants of both genders (age range, 22–59 years). We used some statistical procedures to compare the estimated and perceived hedonic ratings in terms of correlation and concordance in individuals as well as in groups. Our results, although the number of participants was small, showed that the predicted hedonic ratings correlated and agreed well with perceived hedonic ratings toward other tastes, including commercially available beverages (see Figs 4–6), which appears to support the above-mentioned hypothesis and suggests that our formula can be used effectively as an objective evaluation tool regardless of gender or age. However, a significant difference was detected between the two ratings for very aversive sucrose octaacetate (SOA) and very palatable apple juice. Such a difference between the two ratings for SOA and apple juice may have been due to the limitation of the predicted ratings on reaching the maximum hedonic ratings, such as –10 or +10. In our next study, we plan to use a smaller hedonic scale ranging from –5 to +5.

In this study, only one male (age, 40 years) of 12 participants did not show a good correlation between predicted and perceived hedonic ratings. Although his perceived hedonic ratings varied well among different taste stimuli, he showed relatively fewer facial expressions in response to nearly all. One possible reason for this is that he had a beard, which might have diminished the accuracy of the facial analysis. Another possible reason is volitional control of facial expressions. Concerning the gustofacial reflex, Steiner [14] indicated that younger children are unable to suppress well taste-elicited facial responses, suggesting that the gradual maturation of this inhibitory process may control this reflex by means of volition of cortico-pontine connections. Facial expressions are similar to breathing in the sense that automatic or reflex occurrences by brain stem structures can be influenced by volitional control from higher brain centers [30]. A larger study population is needed to determine the rate of appearance of such persons who are apt to depress emotions, e.g., through facial expressions, as seen in the present study.

The regression formula was obtained from a limited number of female students using basic tastes. This might be the reason for the relatively low adjusted R^2^ value (0.451), and for the finding that only “happiness” was a significant predictor of hedonic ratings. A larger study population including both genders and a larger range of ages, along with a wider range of taste stimuli from commercially available foods and beverages therefore needs to be examined. The AI application used in this study for the analysis of facial expressions is available only in Japan. An unexpected result was that this AI application displayed sadness exclusively rather than disgust emotions for facial expressions induced by aversive taste stimuli, such as 5% NaCl, 0.01% citric acid, and 0.02% QHCl; this may be dependent on the AI application. Meanwhile, the present results that happiness and sadness appeared dominantly, and that fear and surprise were correlated, may be related to a recent study by Wang et al. [31] reporting that confusion occurred between fear and surprise as well as between disgust and anger, whereas happiness and sadness were unique, on an emotion recognition test. Facial emotions and scores would be classified differently with different accuracies by a different algorithm, such as FaceReader, iMotions, or Microsoft Face API [32]. This is the most important question to be clarified, and we are now planning to use other AI applications in our next study to examine possible differences. If the same AI is used throughout the experiments, essentially no problem would occur, as in the present study.

Regardless of these limitations, to our knowledge, this is the first study to demonstrate that hedonic ratings can be well predicted by a formula derived from multiple regression analysis of facial expressions obtained using AI software. Another important point is that this procedure is quite easy and the outcomes from the AI application are given very rapidly after uploading a single image. This technique could be expected to be utilized in different situations, such as consumer surveys and new product evaluations, and would be particularly important for those who cannot speak or communicate with others, such as neonates, weaning and/or newly weaned infants, and older persons suffering from dementia.

## Supporting information

S1 Table Raw data for Fig 1. (XLSX)

S2 Table Raw data for Figs 4 and 5. (XLSX)
