# Correction: Analysis of facial expressions in response to basic taste stimuli using artificial intelligence to predict perceived hedonic ratings

**DOI:** 10.1371/journal.pone.0258707

**Published:** 2021-10-12

**Authors:** Takashi Yamamoto, Haruno Mizuta, Kayoko Ueji

The data for D:f(42) in S2 Table are incorrect. Please view the correct S2 Table below.

There is an error in the regression formula, which appears in the Results section and in the Supporting Information file S2 Table. The first coefficient should have been 0.242 instead of 0.024. The correct formula is: Hedonic rating = 0.242 × surprise/fear + 0.138 × happiness + 0.097 × neutral + 0.055 × disgust + 0.021 × sadness + 0.031 × anger– 0.584 × embarrassment − 8.943.

## Supporting information

S2 Table Raw data for Figs 4 and 5. (XLSX)
